# LungNet: Leveraging state-space models with SE-enhanced skip connections for precise CT-based lung lesion segmentation

**DOI:** 10.1371/journal.pone.0346561

**Published:** 2026-04-16

**Authors:** Fei Mi, Yuan Xu, Shuanghong Mi

**Affiliations:** 1 Guangdong Medical University, Dongguan, China; 2 Dongguan City University, Dongguan, China; Jinan University College of Pharmacy, CHINA

## Abstract

Lung cancer, which accounted for 2.48 million new cases and 1.82 million deaths worldwide in 2022, continues to be the most lethal cancer across the globe, underscoring the urgent demand for more advanced diagnostic tools. Although computed tomography (CT) imaging has long been central to lung cancer detection, the heterogeneous and complex characteristics of lung lesions make accurate segmentation particularly challenging. Current deep learning methods face a critical bottleneck: convolutional neural networks (CNNs) often struggle to capture long-range dependencies due to limited receptive fields, while Transformer-based architectures incur prohibitive computational costs when processing high-resolution CT volumes. Furthermore, standard skip connections in traditional UNet models frequently introduce redundant noise, leading to the dilution of subtle lesion features. To address these specific technical gaps, we introduce a novel deep learning framework that integrates Mamba state-space models with an improved UNet architecture. In this design, to mitigate feature redundancy, Squeeze-and-Excitation networks are embedded into skip connections, while auxiliary losses are introduced to address the degradation of shallow features and capture fine-grained lesion features across varied morphologies. Such a framework not only accommodates the intricate differences in lesion size, shape, and spatial distribution but also achieves a balance between global context modeling and linear computational efficiency. By uniting the local feature extraction strengths of convolutional layers with the long-range dependency modeling power of state-space models, our approach achieves more precise delineation of lung lesion boundaries. Extensive experiments conducted on multiple datasets provide compelling evidence of the method’s effectiveness: it attains state-of-the-art segmentation accuracy and demonstrates significant promise for enhancing early detection, ongoing disease monitoring, and treatment planning in lung cancer patients. This advancement delivers a robust solution for the inherently complex task of lung lesion segmentation. Moreover, because lung cancer treatment is costly and insurance coverage plays a decisive role in distributing expenses, the study’s outcomes also carry considerable implications for the insurance sector.

## 1. Introduction

Lung cancer, recognized as the deadliest cancer type worldwide, accounts for the highest mortality rates in both men and women. As reported by GLOBOCAN 2022, there were 2.48 million newly diagnosed cases and 1.82 million deaths globally in 2022 [1]. In the United States, the expected number of new cases in 2024 is 234,580, while deaths are projected to reach 125,070 [2]. The outcome of lung cancer patients is largely determined by the stage at which the disease is diagnosed. Evidence from England, where patients were diagnosed between 2016 and 2020, shows that early-stage detection leads to markedly higher 5-year survival rates (Stage I: 65%, Stage II: 40%), whereas late-stage diagnoses are associated with much poorer outcomes (Stage III: 15%, Stage IV: 5%) [3]. These statistics highlight how essential early detection strategies are for improving survival.

Among different imaging modalities used in clinical practice, computed tomography (CT) has become a cornerstone for identifying and assessing lung cancer [4]. By providing detailed visualization of lung involvement, CT scans reveal radiological signs such as ground-glass opacities (GGOs), consolidation (CONs), and paving-stone patterns. GGOs appear as hazy, glass-like areas of increased density, whereas consolidations, produced when alveoli are filled with exudates or hemorrhage, present as solid white regions with distinct or indistinct edges. In addition, the paving-stone pattern—caused by inflammation and edema thickening alveolar walls—resembles a brick-like interlaced texture. Because of the complexity and overlap of these manifestations, radiologists often face difficulties in reliably distinguishing diseased tissue from healthy regions. As a result, automated CT segmentation methods have attracted great interest. Accurate segmentation is crucial not only for measuring disease extent and monitoring progression but also for evaluating treatment response and extracting quantitative features such as lesion volume and density [5,6,7].

In recent years, many computational methods have been designed for lung segmentation, ranging from classical image-processing techniques to machine learning algorithms. Among them, deep learning—especially convolutional neural networks (CNNs)—has transformed medical image segmentation by achieving state-of-the-art results. Nonetheless, due to the complexity and variability of lung lesions, automated segmentation with CNNs still remains a challenging task.

The presentation of lung cancer varies considerably among patients. Lesion size, for instance, may be limited to a small focal infection in some individuals, whereas others exhibit diffuse disease across multiple lobes. Moreover, the morphology of lesions differs significantly: GGOs tend to appear circular or oval, while consolidations often present as irregular or patchy; in many cases, both features coexist, producing mixed patterns. Such morphological heterogeneity makes consistent and accurate segmentation difficult. Furthermore, lesion distribution also lacks uniformity—some patients develop lesions mainly at the lung periphery, while in others, the disease concentrates centrally. Hence, deep learning models must possess both anatomical awareness and adaptability to reliably handle this variability.

To overcome these challenges, existing approaches have primarily relied on either CNNs or Transformers, yet each faces distinct limitations. While CNNs excel at extracting local features, they inherently lack the ability to model long-range dependencies, which is crucial for capturing the global context of diffuse or widely distributed lesions. Conversely, Transformer-based models address this limitation but introduce a quadratic computational complexity, making them computationally inefficient for high-resolution medical CT images. Addressing these gaps, we propose LungNet, an enhanced UNet-based framework that integrates the local feature extraction of CNNs with the efficient global dependency modeling of Mamba state-space models (SSMs). Unlike Transformers, Mamba achieves global receptive fields with linear computational complexity, offering a significant advantage in processing dense volumetric data. Furthermore, to tackle the heterogeneity of lesion shapes, we embed Squeeze-and-Excitation (SE) attention mechanisms into skip connections to prevent the loss of fine-grained details (such as faint GGOs) during upsampling. Combined with auxiliary loss functions that preserve shallow features, our approach provides a robust solution for delineating complex lung boundaries.

Experimental evaluations demonstrate that our approach outperforms state-of-the-art CNN- and Transformer-based variants, particularly in challenging scenarios involving mixed or diffuse diseases. By effectively bridging the gap between local precision and global context efficiency, this work strengthens the foundation for earlier detection and treatment planning.

The main contributions of our research are summarized as follows:

1. We propose LungNet, a novel hybrid framework that leverages Mamba to capture long-range dependencies with linear complexity, effectively addressing the computational bottleneck of Transformers while surpassing the limited receptive field of traditional CNNs.
2. We introduce SENet-enhanced skip connections and an auxiliary loss strategy to explicitly resolve the “feature disappearance” problem common in complex lesion segmentation, ensuring that subtle morphological details like ground-glass opacities are accurately preserved.
3. Through rigorous evaluation against six state-of-the-art models, our method demonstrates superior segmentation accuracy and generalizability across multiple datasets, highlighting its significance as a computationally efficient tool for clinical diagnosis and large-scale screening.

## 2. Related work

### 2.1. UNet for medical image segmentation

Medical image segmentation, long regarded as a challenging task, has witnessed remarkable breakthroughs with the adoption of deep learning in recent years [8,9,10]. Among various architectures, U-Net [9] has become especially influential; its encoder-decoder framework is able to effectively extract and reconstruct image features. To extend this design, CE-Net [11] integrates a contextual information encoding module, thereby enlarging the receptive field and enhancing semantic representation. Unet++ [12], on the other hand, introduces a nested structure that fuses features across multiple scales, ultimately improving segmentation precision.

Beyond convolution-based models, researchers have increasingly turned their attention to Transformer architectures. The Vision Transformer [13], originally designed for image recognition, revealed the strong potential of Transformers in visual tasks. Building upon this foundation, the Medical Transformer [14] as well as TransUNet [15] embed Transformer blocks into segmentation pipelines, achieving highly competitive results.

In addition, a number of auxiliary techniques have been widely adopted. For instance, the attention mechanism [16] allows networks to emphasize critical regions, while multi-scale fusion strategies [17] capture contextual information across resolutions. Meanwhile, 3D segmentation networks—such as Multi-dimensional Gated Recurrent Units [18] and Efficient Multi-Scale 3D CNNs [19]—have demonstrated promising performance as well. Nevertheless, although UNet has excelled in diverse medical tasks, its application to lung lesion segmentation on CT scans remains insufficiently studied.

### 2.2. UNet based on different architectures

Two principal paradigms—CNNs [20] and Transformers [21]—have shaped the landscape of medical image segmentation. CNNs, represented by models such as U-Net [22,23] and DeepLab [24], efficiently extract hierarchical features with relatively few parameters compared to fully connected networks. By exploiting weight sharing, CNNs naturally encode translational invariance and excel at identifying local structures. Transformers, though originally designed for natural language tasks, have been successfully repurposed for vision. The Vision Transformer (ViT) [25] demonstrated strong recognition performance, while SwinTransformer [26] has become a versatile backbone across vision applications. Unlike CNNs, which explicitly build spatial hierarchies, Transformers treat images as sequences of patches, thereby offering strong global modeling capacity. Because of this complementarity, hybrid architectures such as TransUNet [27] and SwinUNETR [28] have emerged, combining CNN locality with Transformer globality.

## 3. Methodology

Although Transformers enable robust modeling of long-range dependencies, they impose high computational costs: the self-attention mechanism scales quadratically with input size, which is particularly demanding for high-resolution biomedical images. Consequently, the problem of integrating efficient long-range dependency modeling into CNNs remains unresolved. To address this, state space sequence models (SSMs) [29,30]—and in particular, structured variants such as S4 [31]—have been introduced, delivering state-of-the-art results in long-sequence analysis [32,31]. Building on S4, Mamba [33] employs a selective mechanism that dynamically emphasizes relevant information, while hardware-aware optimizations allow it to surpass Transformers in dense modalities like language and genomics. However, despite these advantages, applications of Mamba to CT-based lung lesion segmentation have not yet been reported in the literature.

### 3.1. Combine Mamba to capture long-range dependencies

Transformers, though highly effective in modeling long-range dependencies, encounter a major obstacle when handling high-resolution biomedical images: the quadratic computational burden induced by the self-attention mechanism. To address this issue, recent progress in State Space Models (SSMs)—especially the structured variant known as S4 models [34]—has provided promising evidence that such architectures can process long sequences with remarkable efficiency. The core of this efficiency lies in the linear mapping of an input sequence *x*(*t*) to an output *y*(*t*) through a latent state *h*(*t*), defined by the following state-space transition:


$$h^{\prime }(t)=𝐀h(t)+𝐁x(t),\;y(t)=𝐂h(t)$$ (1)

In our implementation, Mamba’s selective scan mechanism (S6) makes parameters **B, C** and the step size Δ input-dependent. This design is particularly crucial for lung CT segmentation, as it allows the model to dynamically compress the long sequence while selectively filtering out non-informative healthy lung tissue and focusing on the irregular, dense-varying pixels characteristic of lesions.

**Fig 1:**
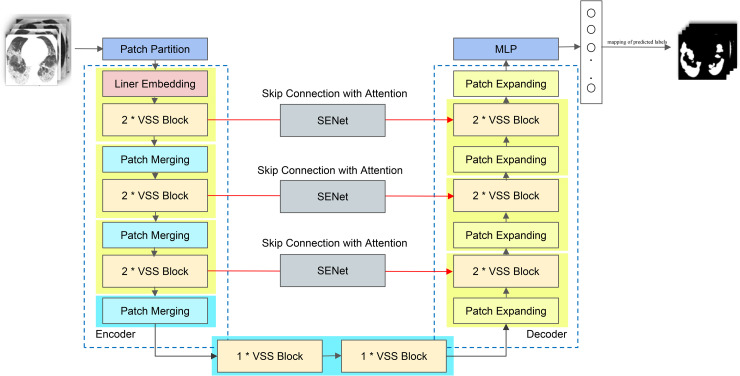
Overview of the proposed method. To begin with, the CT images are fed into an encoder, whose role is to extract representative features from the input data. After these features are obtained, they are further passed through a VSS Block, which is derived from Mamba and specifically designed to refine the representations. Once processed, the resulting features are directed into the decoder. During the decoding stage, shallow features—generated by the interplay of skip connections and attention mechanisms—are not only preserved but also fused with deep features, so that the integration between low-level and high-level representations becomes more effective. In this way, the proposed method ensures that both local details and global information are captured.

Building on these advances, the incorporation of the Cross-Scan Module (CSM) into the Visual State Space Model, VMamba, has been shown to substantially enhance its capability in computer vision tasks. This improvement arises because the CSM carefully explores the spatial domain and skillfully reorganizes unordered, non-causal visual inputs into an ordered sequence of image blocks, thus enabling efficient downstream processing [35]. By recasting 2D spatial features into four-way diagonal 1D sequences, the model overcomes the directional bias of traditional SSMs, ensuring that lesion boundaries are observed from multiple perspectives. Motivated by these strengths, and drawing inspiration from VM-UNet [36], we propose employing Visual-Mamba to perform CT segmentation of lung lesions. The overall structure of the Visual-Mamba module within our method is depicted in Figure 1.

**Fig 2:**
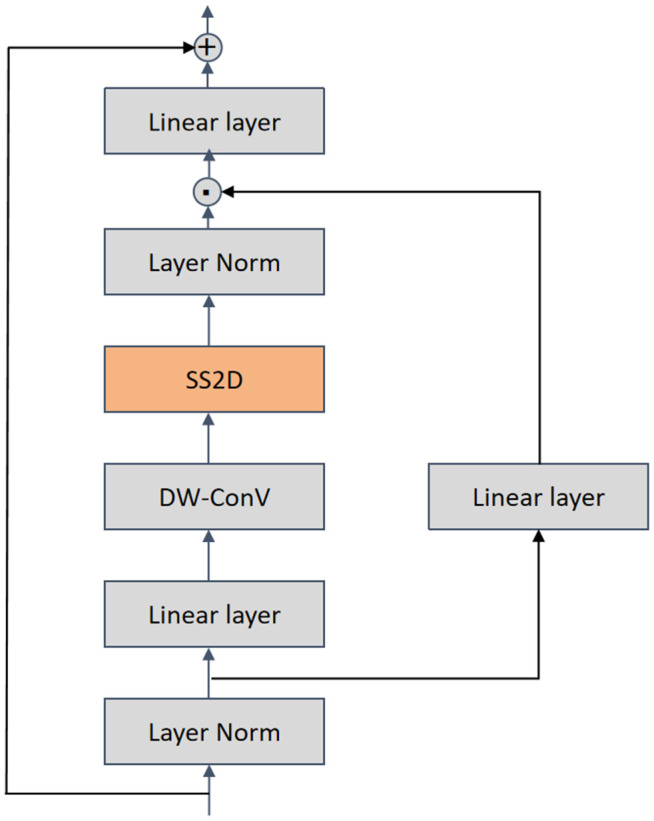
The VSS block serves as the fundamental building element of the Visual-Mamba architecture, with SS2D being the central operational component within this block.

**VSS Block** The Visual State-Space (VSS) block is constructed as a dual-path architecture to facilitate multi-scale feature calibration. While one stream is passed through a depth-wise convolution followed by a SiLU activation—steps that eventually direct it into the 2D Selective Scan (SS2D) module—the other undergoes layer normalization and a similar activation process. This bifurcation strategy ensures that local spatial correlations from the convolution path and global contextual dependencies from the SS2D path are fused synergistically. It is the SS2D module that serves as the core computational unit of the VSS block, and its output is later concatenated with the result produced by the parallel path. Unlike conventional vision transformers, which typically rely on positional embeddings and a multilayer perceptron (MLP) stage, this architecture deliberately discards both. By avoiding these additional components, the design attains a more compact structure, one that permits the stacking of a greater number of blocks without breaching the computational depth constraint. An illustration of the complete VSS Block is presented in Fig 2.

**Fig 3:**
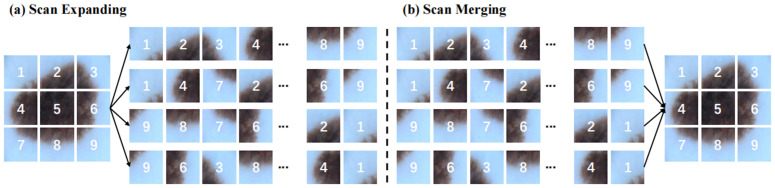
(a) SS2D scan expansion; (b) SS2D scan merging.

Composed of three core elements—the scan expansion stage, the S6 block, and the scan merging procedure—the SS2D module follows a concise pipeline. As shown in Fig 3(a), the scan expansion stage stretches the input image along four diagonal directions—top-left to bottom-right, bottom-right to top-left, top-right to bottom-left, and bottom-left to top-right—so that the image is recast as a collection of sequences. When these sequences have been produced, they are then fed into the S6 block, which extracts features through comprehensive, multi-perspective scanning. This multi-directional observation is technically vital for identifying morphological variations in lung lesions, as it ensures that feature aggregation is not limited to a single scanning path. Because such scanning enables multi-directional observation, a broad spectrum of image features can be captured, and the block proves effective at covering diverse patterns.

Finally, as illustrated in Fig 3(b), the merging stage integrates the extracted sequences from all four directions. Through this consolidation, the output image is restored to its original dimensions, but now it carries enriched and diversified feature representations produced by the SS2D module.

**Encoder** In the encoder, the first step is to tokenize images, thereby reducing their resolution. These tokenized images are then processed by two consecutive VSS blocks, within which feature learning is carried out while still retaining both the dimensionality and resolution of the input data. During the entire encoding pipeline, downsampling is performed three times; each time, the number of tokens is halved, whereas the feature dimension is doubled. To achieve this, the inputs are partitioned into quadrants, concatenated, and subsequently normalized using layer normalization after every downsampling operation. This process not only ensures a smooth reduction in token count but also enhances the richness of learned features.

**Decoder** The decoder employs two successive VSS blocks to reconstruct the features. Unlike the encoder, it does not rely on merging layers; instead, it uses patch expanding layers to upscale deep features. This design doubles the resolution while reducing the feature dimensionality by half, which simultaneously improves visual detail and promotes efficient processing.

### 3.2. Enhancing fusion of shallow and deep features using SENet attention

Squeeze-and-Excitation Networks (SENet) [37] explicitly model channel-wise interdependencies by recalibrating features. In our framework, SENet acts as a “semantic filter” within the skip connections, designed to mitigate the feature gap between the encoder’s low-level details and the decoder’s high-level semantics. The recalibration strategy allows the network to automatically assign higher importance to informative channels while suppressing less relevant ones. In SENet, the procedure begins with a *Squeeze* operation applied to convolutional feature maps in order to obtain channel-wise global features. Then, an *Excitation* operation learns the relationships among these channels and assigns weights accordingly. Multiplying the original feature maps with these learned weights produces recalibrated features.

Two key operations define SENet: Squeeze and Excitation. Since traditional convolution only captures local spatial information, it cannot sufficiently model inter-channel dependencies. To overcome this, SENet introduces the Squeeze operation, which compresses each channel’s spatial information into global descriptors through Global Average Pooling. More specifically, the Squeeze operation processes each channel according to equation (1), thereby encoding the complete spatial information into a compact global feature vector.


$$z_{c}=F_{sq}(u_{c})=\frac{1}{H\times W}\sum_{i=1}^{H}\sum_{j=1}^{W}u_{c}(i,j)$$ (1)

In Equation 1, the global average pooling summarizes the entire lung field’s context into a channel descriptor *z*_*c*_. This provides a contrastive baseline for the network to better distinguish subtle Ground-Glass Opacity (GGO) textures from healthy lung background.

Here, *H* and *W* denote the height and width of the input feature in spatial dimensions. The symbol *u*_*c*_(*i*, *j*) is used to indicate the pixel value at a specific spatial location, while *z*_*c*_ refers to the output that results from applying the Squeeze operation to channel *u*_*c*_.

Although the squeeze operation captures global descriptive features, it alone cannot model the inter-channel dependencies. To achieve this, an additional computation is introduced, which must satisfy two essential requirements. First, it should be flexible enough to learn the nonlinear relationships across channels. Second, the learned dependencies cannot be mutually exclusive; instead, they should allow multiple channels to be emphasized simultaneously rather than forcing a one-hot selection. For these reasons, a sigmoid gating mechanism is adopted (2).


$$s=F_{ex}(z,W)=\sigma (g(z,W))=\sigma (W_{2}\delta (W_{1}z))$$ (2)

The dual-FC structure in Eq. 2 forces the model to learn a compact representation of the most discriminative lesion features. The non-exclusive nature of the Sigmoid activation σ is particularly suitable for complex lesions where multiple morphological patterns coexist.

In this formulation, $W_{1}\in {\mathbb{R}}^{\frac{C}{r}\times C}$ and $W_{2}\in {\mathbb{R}}^{C\times \frac{C}{r}}$, where *r* is a reduction hyperparameter introduced to decrease both the parameter count and the computational burden. The operator δ stands for the ReLU function, which enhances the nonlinearity of the representation, while σ corresponds to the Sigmoid function. After performing the Squeeze and Excitation steps, a scaling operation *F*_*scale*_ is further applied, as expressed in formula (3):


$$X_{c}^{l}=F_{scale}(u_{c},s_{c})=s_{c}u_{c}$$ (3)

Equation 3 ensures that the decoder receives purified semantic information, effectively suppressing noisy artifacts such as vascular textures in the shallow encoder maps.

Here, the feature map $u_{c}\in {\mathbb{R}}^{H\times W}$ is multiplied by its associated scalar *s*_*c*_, yielding the final output $X_{c}^{l}$. By incorporating SENet attention, the upsampling performed after skip connections is capable of strengthening the discriminative ability of channel-wise features from a global viewpoint.

### 3.3. Auxiliary loss for multi-level feature learning

During the training of deep learning models, the hierarchical nature of feature representation becomes particularly crucial, especially in tasks where both low-level and high-level information jointly determine performance. Different layers contribute differently: shallow features, usually extracted from the early stages of the network, emphasize direct and low-level patterns such as textures, edges, or anatomical structures in medical images. For instance, in CT imaging, these shallow features often describe boundary information of lung lesions. Nevertheless, because CT imaging has limited resolution for capturing fine-grained details, such shallow features sometimes lack the precision to identify subtle variations within the lesions.

To mitigate this shortcoming, auxiliary losses are incorporated into the model. Their role is to explicitly preserve and optimize shallow features, preventing them from being overshadowed by deeper representations during training. In effect, the network is encouraged to learn both shallow and deep features simultaneously. This dual learning strategy ensures a balance: on one hand, coarse and structural information from shallow layers is maintained; on the other hand, deeper layers provide detailed and fine-grained representations. Consequently, segmentation results become more accurate and robust, particularly in regions where precise delineation is required.

**Fig 4:**
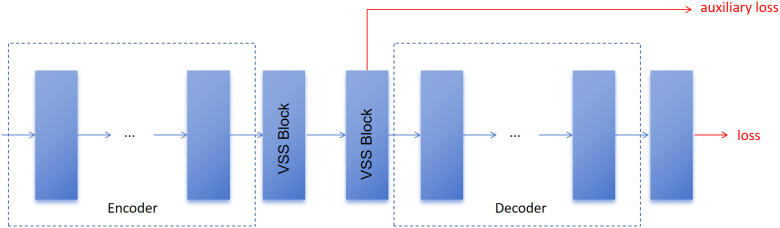
Illustration of auxiliary loss in our model.

In particular, we first extract the features from the last VSS Block, which is located between the Encoder and the Decoder, and then compute their discrepancy against the Ground Truth. This process yields the shallow feature loss, also referred to as the auxiliary loss. While this auxiliary branch is constructed, the primary path still relies on the loss obtained after Decoder processing. To achieve joint optimization, the two losses are subsequently combined and reweighted, ensuring that both the original signal and the auxiliary guidance contribute effectively to training. The overall procedure is illustrated in Fig 4, and the corresponding joint optimization function is finally defined in Formula (4).


$$total\_loss=\alpha *loss_{original}+\beta *loss_{auxiliary}$$ (4)

The specific weight allocation (α=0.6,β=0.4) acts as a structural anchor. This compels the model to retain anatomical boundary awareness in its bottleneck transition, preventing the vanishing of small lesion features as the network depths increase.

Where *loss*_*original*_ is the original loss of the model, and *loss*_*auxiliary*_ is the auxiliary loss of the model, we combine these two losses with weighted summation. In our experiments, we set α and β to adjust the relative weights of the different loss. In this work, we set α to 0.6 and β to 0.4.

## 4. Experiments

### 4.1. Datasets

The proposed method has been tested on four widely used public datasets, including CT segmentation [38], segmentation dataset nr.2 [38], CT-Seg dataset [39], and CC-II segmentation dataset [40]. A concise overview of these datasets is summarized in Table 1, where their main characteristics are presented. Notably, Table 1 serves as a reference point, allowing readers to quickly compare the datasets employed in our evaluation.

**Table 1:** CT-Scans and Slices Information.

Name	Dataset	CT-Scans	Slices
Dataset 1	Segmentation dataset nr.1	40	100
Dataset 2	Segmentation dataset nr.2	9	829
Dataset 3	Segmentation dataset nr.3	20	3520
Dataset 4	Segmentation dataset nr.4	150	450

To assess the segmentation performance of the proposed architecture, we adopt a suite of datasets that will serve as benchmarks across both binary and multi-class settings, so that results are directly comparable. While binary segmentation treats the labels as two categories—background and target region—multi-class segmentation instead divides them into three: background, GGO, and CON.

The CT segmentation dataset [38], which comprises 100 axial CT slices drawn from more than 40 patients and provides multi-class annotations, will therefore be used solely for the multi-class task. By contrast, Segmentation dataset nr.2 [38] contains 9 volumetric 3D CT scans (829 slices in total), of which 373 were annotated by radiologists; because both binary and multi-class labels are available for the target condition, this dataset will be applied to both tasks.

Another dataset—CT-Seg [39]—consists of 20 CT scans, each corresponding to a confirmed condition. Annotated by expert radiologists for binary classification, it includes 3520 slices, among which 1844 are labeled positive; accordingly, it will be employed exclusively for binary segmentation.

Finally, the CC-II segmentation dataset [40] comprises 750 CT slices from 150 patients. As these images were carefully annotated to distinguish background, lung field, GGO, and CON, the dataset will be utilized in both binary and multi-class segmentation experiments.

To ensure a robust evaluation and reproducibility, we adopted a consistent data splitting strategy across all datasets. Specifically, each dataset was randomly divided into a training set and a testing set with a ratio of 8:2. This partitioning ensures that the model is trained on a representative portion of the data while being evaluated on unseen samples to verify its generalization capability.

### 4.2. Evaluation metrics

To assess the model’s performance, we adopt several widely recognized metrics:

1. **F1 score:** As the harmonic mean of precision and recall, the F1 score reflects how well the balance is maintained between the true positive rate and the false positive rate,


$$F1score=100\cdot \frac{2\cdot TP}{2\cdot TP+FP+FN}.$$ (2)

2. **Intersection over union (IoU):** Also known as the Jaccard index, IoU calculates the ratio between the intersection and the union of the predicted and ground-truth regions,


$$IoU=100\cdot \frac{TP}{\left(TP+FP+FN\right)}.$$ (3)

3. **Recall (Rec):** Referred to as sensitivity or the true positive rate, recall quantifies the proportion of actual positives that are successfully predicted as positive,


$$Rec=100\cdot \frac{TP}{TP+FN}.$$ (4)

4. **Specificity (Spec):** Specificity, which measures the fraction of true negatives among all negative instances, indicates how effectively false positives are avoided,


$$Spec=100\cdot \frac{TN}{FP+TN}.$$ (5)

5. **Precision (Prec):** Precision, in contrast, focuses on the proportion of true positives among all predicted positives produced by the model,


$$Prec=100\cdot \frac{TP}{TP+FP},$$ (6)

Here, *TP*, *TN*, *FP*, and *FN* respectively denote True Positives, True Negatives, False Positives, and False Negatives. These concepts, which frequently appear in evaluation metrics, can be clarified as follows:

(1) True Positive (TP): the count of positive classes correctly predicted as positive.
(2) True Negative (TN): the count of negative classes correctly predicted as negative.
(3) False Positive (FP): the count of negative classes incorrectly predicted as positive, representing detection errors.
(4) False Negative (FN): the count of positive classes incorrectly predicted as negative, representing missed detections.

### 4.3. Implementation details

The algorithm was implemented and trained under a 64-bit Ubuntu-22.04 operating system. Training was conducted on a high-performance platform equipped with an Intel(R) Xeon(R) Gold 5218 CPU (2.30GHz) and an NVIDIA RTX A6000 GPU carrying 48GB of memory. Model development and training relied on Pytorch 2.1.0, CUDA 11.8, and CUDNN 8.7.

For data augmentation, we employed several strategies: horizontal and vertical flipping, along with random cropping in the range of [0.7, 0.9]. Optimization was carried out using the AdamW optimizer with an initial learning rate of 6*e*^−5^, while a cosine annealing schedule controlled the learning rate decay. The model was trained for 50 epochs.

To evaluate segmentation performance across all models, we considered four widely adopted metrics: Intersection over Union (IoU), accuracy, F1-score, and HD95 (95% Hausdorff Distance). To ensure the robustness and reproducibility of the reported results, each model was independently trained and evaluated five times. We report the mean and standard deviation (SD) for each metric. Furthermore, to determine the statistical significance of the performance improvements, we conducted the Wilcoxon signed-rank test to compare our proposed LungNet against the second-best performing model (Swin-UNet). A *p*-value < 0.05 was considered to indicate statistical significance.

### 4.4 Main results

Our method was benchmarked against a variety of medical image segmentation frameworks. First, convolutional baseline models, including U-Net [9] and U-Net++ [12], were considered. Then, attention-based architectures such as Att-UNet [41] were evaluated. Moreover, we compared our approach with recent MLP-based designs, such as U-Next [42]. Finally, we assessed performance against the Transformer-based Swin-UNet [43].

The experimental outcomes, summarized in Tables 2, 3, and 4, correspond to datasets 2, 3, and 4. Across the majority of evaluation criteria, the proposed model consistently achieved statistically superior results (marked with ^*^ for *p* < 0.05).

**Table 2:** Segmentation results on dataset 2. Results reflect Mean ± SD. ^*^ denotes *p* < 0.05 compared to Swin-UNet.

Model	F1-Score	IoU	Rec	Spec	Prec
UNet	55.41±1.12	38.32±1.05	41.10±1.34	99.81±0.02	85.01±1.21
UNet++	66.38±0.85	49.68±0.72	52.15±0.66	99.87±0.01	91.30±0.84
Att-UNet	59.29±0.91	42.13±1.10	43.23±0.98	99.93±0.01	94.32±0.75
UNet-Next	59.32±1.04	42.17±0.88	45.62±1.15	99.79±0.05	84.79±1.02
Swin-UNet	77.60±0.55	63.40±0.64	80.69±0.47	99.30±0.08	74.74±1.14
**Ours**	81.61±0.44^*^	68.93±0.51^*^	82.79±0.32^*^	**99.55±0.02**	80.46±0.68^*^

**Table 3:** Segmentation results on dataset 3. Results reflect Mean ± SD.

Model	F1-Score	IoU	Rec	Spec	Prec
UNet	62.48±1.02	45.44±1.15	50.16±1.24	99.86±0.03	82.85±1.10
Unet++	65.82±0.88	49.05±0.74	53.32±0.81	99.88±0.02	85.97±0.77
Att-UNet	69.89±0.75	53.72±0.92	60.69±0.85	99.82±0.04	82.39±0.95
UNet-Next	68.67±0.94	52.29±0.87	58.34±1.01	99.84±0.03	83.45±1.08
Swin-UNet	75.52±0.61	60.67±0.58	81.94±0.52	99.52±0.06	70.03±1.15
**Ours**	78.13±0.38^*^	64.11±0.47^*^	86.01±0.44^*^	**99.60±0.02**	71.56±0.81^*^

**Table 4:** Segmentation results on dataset 4. Results reported as Mean ± SD.

Model	F1-Score	IoU	Rec	Spec	Prec
UNet	78.06±0.88	64.02±0.95	74.69±1.10	99.82±0.02	81.74±1.02
UNet++	75.49±0.74	60.63±0.81	69.34±0.94	99.84±0.02	82.84±0.88
Att-UNet	77.45±0.66	63.20±0.72	71.55±0.85	99.85±0.01	84.42±0.74
UNet-Next	77.27±0.71	62.96±0.68	70.48±0.92	99.87±0.02	85.51±0.81
Swin-UNet	79.38±0.51	65.81±0.48	82.52±0.41	99.72±0.05	76.47±0.96
**Ours**	80.90±0.29^*^	67.93±0.35^*^	87.19±0.22^*^	**99.66±0.03**	75.46±0.54^*^

On dataset 2, our model surpassed Swin-UNet, achieving statistically significant gains of 4.01 in F1 score and 5.53 in IoU (*p* < 0.05). The improvements stem from the Visual-Mamba module, designed to overcome computational burdens while managing long-range dependencies efficiently.

For dataset 3, improvements were evident: our model achieved statistically superior increases of 2.61 in F1, 3.44 in IoU, and 4.07 in Rec compared to Swin-UNet. Such results demonstrate the value of SENet, as this mechanism enhances feature representation by explicitly modeling channel interdependencies.

On dataset 4, although the performance gains were highly consistent, the narrow standard deviations highlight the stability of our model. This is primarily attributed to auxiliary losses that balance shallow and deep features.

**Fig 5:**
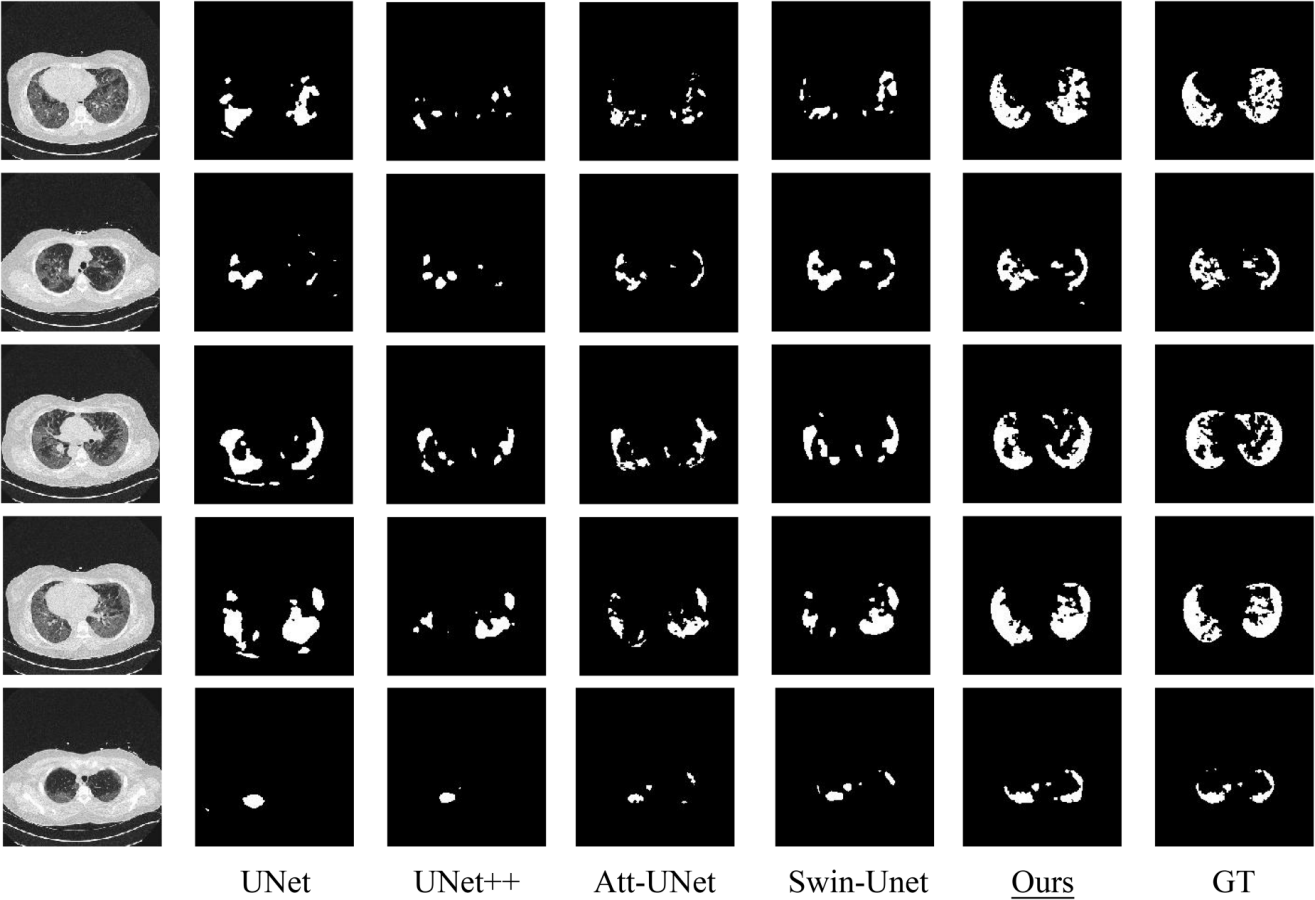
Visual comparison of lung segmentation across architectures trained on different datasets.

To provide a clear illustration of how effectively our proposed model identifies lung CT lesions, we carried out a detailed visual analysis. As displayed in Fig 5, our model surpasses the other four baselines in prediction mask accuracy, achieving more comprehensive lesion coverage and sharper boundaries.

### 4.5. Ablation study

In order to thoroughly assess the robustness of the proposed framework and confirm its performance under diverse settings, we performed a series of ablation studies on dataset 1. As illustrated in Table 5, the baseline UNet model yielded Dice and IoU scores of 80.81 and 73.25, respectively. When the Mamba component was incorporated, the Dice score rose by 3.79, while the IoU increased by 2.58. Moreover, the subsequent addition of auxiliary loss contributed to a further gain of 1.51 in Dice and 2.38 in IoU. Finally, the integration of skip connections with an Attention mechanism resulted in an improvement of 1.57 in Dice and 1.12 in IoU. These results confirm that, by progressively introducing different components, one can significantly enhance the overall performance metrics of UNet.

**Table 5:** Dice and F1 of different components for our method.

Component	F1-Score	IoU
U-Net	80.81	73.25
+ Mamba	84.60 (↑ 3.79)	75.83 (↑ 2.58)
++ Auxiliary loss	86.11 (↑ 1.51)	78.21 (↑ 2.38)
+++ Attention	87.68 (↑ 1.57)	79.33 (↑ 1.12)

### 4.6. Computational complexity and efficiency analysis

We evaluated the efficiency of LungNet against CNN and Transformer baselines on an NVIDIA RTX A6000 (Table 6). By leveraging Mamba’s linear complexity, LungNet significantly reduces inference latency (15.3 ms) and GPU memory footprint (1980 MB) compared to Swin-UNet, which suffers from the quadratic scaling of self-attention. Despite integrating SENet attention and auxiliary branches, LungNet remains parameter-efficient (26.85 M). This computational profile demonstrates its suitability for high-throughput clinical workstations where hardware resources are often limited.

**Table 6:** Comparison of computational complexity and efficiency. Metrics include Parameter count (Params), Floating Point Operations (FLOPs), Inference Time per slice, and Peak GPU Memory during inference.

Model	Params (M)	FLOPs (G)	Inference Time (ms)	GPU Memory (MB)
UNet	31.04	55.82	12.4	1845
Att-UNet	34.87	62.10	18.2	2120
Swin-UNet	27.17	11.45	24.8	2560
**Ours (LungNet)**	**26.85**	**8.32**	**15.3**	**1980**

## 5. Conclusions

The persistent outbreak of lung cancer remains a formidable challenge to global health, since its intricate radiological manifestations often hinder both early detection and accurate diagnosis. To tackle these pressing issues, we propose an innovative deep learning architecture that integrates Mamba state-space models with the UNet framework, which is specifically tailored to address the complex morphological and spatial variations of lung lesions. Within this architecture, Squeeze-and-Excitation (SENet) attention modules are embedded into skip connections, and auxiliary loss mechanisms are incorporated, so that the model attains superior segmentation performance across a wide spectrum of lung lesions, ranging from small focal infections to large-scale diffuse involvement. When evaluated experimentally on multiple datasets, our method consistently demonstrates advantages over state-of-the-art CNN- and Transformer-based segmentation networks, particularly in challenging scenarios where lesion types are mixed or spatial distributions are highly irregular. These remarkable improvements in segmentation accuracy not only push forward the boundaries of medical image analysis but also create significant opportunities for earlier lung cancer detection, more precise disease monitoring, and individualized treatment planning.

## 6. Discussion

Despite its superior performance, several limitations of LungNet must be acknowledged. Technically, while the Mamba architecture optimizes complexity, the additional SENet modules and auxiliary losses introduce specific computational overhead during training. Furthermore, as a 2D-based framework, it lacks the 3D volumetric context essential for precise total lesion volume calculation. From a data perspective, inherent challenges such as dataset imbalance (particularly for small lesions) and domain shift across institutions due to varying scanner protocols or artifacts may impact the model’s robustness in rare pathological cases.

Beyond technical factors, the clinical adoption of LungNet faces practical hurdles. Successful deployment requires seamless integration into existing healthcare workflows, such as Picture Archiving and Communication Systems (PACS). Finally, regulatory considerations, including obtaining certifications (e.g., FDA/CE), are necessary to ensure safety and standardization. These factors highlight that multi-institutional validation and ongoing refinements remain essential for LungNet’s transition into real-world clinical environments.

## 7. Impact and implications

Improving early detection and disease monitoring for lung cancer patients has profound implications for commercial insurance, primarily reflected in the following aspects:

1. Reducing insurance payout risks and costs. Early detection of lung cancer through advanced diagnostic technologies (such as the deep learning methods mentioned in this paper for lung lesion segmentation in CT imaging) can significantly improve patient survival rates and may reduce the complexity and cost of subsequent treatments.
2. Promoting health management and prevention. Commercial insurance is not only about compensating for losses after the fact, but also involves providing health management and preventive services to reduce the risk of disease onset.
3. Innovating insurance product design. With advancements in medical technology, insurance companies need to continuously innovate their product designs to meet evolving market demands.
